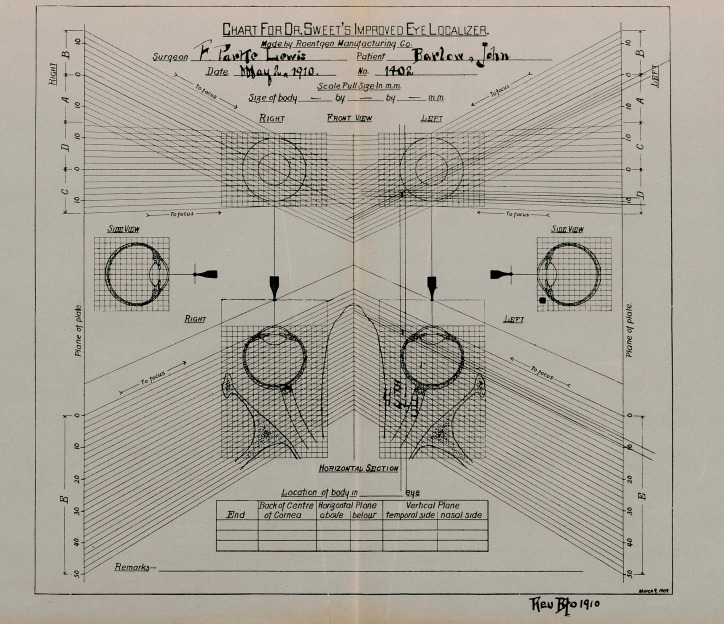# Letters to Physicians

**Published:** 1911-09

**Authors:** George M. Gould

**Affiliations:** Ithaca, N. Y.


### Letters to Physicians

### IV. Heterophoria Superstition

By GEORGE M. GOULD. M.D.
Ithaca, N. Y.

IN looking over and thinking of the case-histories of the patients of the last several years, I conclude that about fifty per cent. of the oculists of civilised countries utterly ignore their patients' heterophoria, that is, the balance or imbalance of the muscles. (When the heterophoria has developed into strabismus the interest, if it exists, is surgical only). Perhaps twenty-five per cent. work at the heterophoria problem through prisms incorporated in the lenses of the ametropic correction, or by prism-gymnastics, or by decentering lenses. A part of the remaining twenty-five per cent. proceed to tendon-tailoring at once if the patients' trust permits and his bank-book authorises. The residuum is so perplexed and uncertain that it flirts with the methods of one or the other classes, at last, from caution, securing safety by following the suit and lead of the fifty per cent. ignorers.

It is thus clear that in the ophthalmological specialty there is no unity, that nearly every authority differs from the others in advice and practice, and that anything approximating science is a ludicrous nonattainment. This is cruelly demonstrated by the fact that every oculist changes the glasses of his predecessor, and that if one-hundred should prescribe for the same patient each prescription would be utterly different from every other.

Patients come to me every day who have had the tendons of the ocular muscles clipped, or who are wearing prisms in their lenses, prisms of all degrees, and at all axes, for muscle-imbalance of all kinds, many of the latter being increased instead of neutralised by the reverse of the indicated prisms. And, of course, without relief of one of their old eyestrain symptoms. Most of them have been driven to coffee, tea, alcohol or drugs, to diet-crankery, to sanitariums, to (other than ocular) surgery, to "rest-cures," to gastrologists and neurologists, to chronic invalidism, to Eddyism, osteopathy, to despair.

Why? Because the tendon-tailors and prism-orderers have ignored, or disbelieved in, these truths long ago and frequently demonstrated:

1. In five minutes, by prism-gymnastics, one can give any patient almost any (temporary and changing) exophoria he pleases,—demonstrating the innervational not muscular (or tendon) nature of the anomaly, and the silly fallacy both of the prism-wearing and of tenotomy, and the like.

2. The fashionable professional, or textbook theories of the origin of heterophoria, are all utterly false. There is no law of the relation of heterophorias to tendon-insertions, or to convergence, or to myopia and hyperopia. The sources of heterophoria are astigmatism and anisometropia, with other contributing factors such as amblyopia, the writing posture, righteyedness and lefteyedness as related to righthandedness and lefthandedness, and the like,—in a word, to "Eyestrain" as properly understood, i. e., to the exclusion of the anatomy or powers of the muscles themselves.

3. No tendon-tinkering ever of itself cured any heterophoria, unless that of murdered eyes.

4. No prisms in the lenses worn ever of itself cured heterophoria.

5. What cured the heterophoria, if it was cured, was the extinction of eyestrain by the adequate correction of the ametropia.

6. Not one in a hundred patients have their ametropia rightly corrected. The reasons for this failure to diagnosticate and prescribe for ametropia would require a big book to set forth. But the chief are:

1. Nine-tenths of oculists have not the tools whereby the most expert man in the world could diagnose ametropia.

2. The prescribing of glasses requires a skill, and patience, and self-sacrifice possessed by but few men.

3. The opticians' work by bungling and maladjusting will usually succeed in making even correct lenses—do more harm than good.

4. The vanity and carelessness of uninstructed and unwatched patients will also usually render good or bad spectacles of little good and often of evil.

Only an endowed, scientific, and thoroughgoing Refraction School will be able to rectify all these evils through educated young men filled with the living religion of preventive medicine.

Of hundreds of cases that illustrate the verity of the foregoing I append two:

A woman, aged 39, came to me September 22, 1910, having had headaches since a child. She has submitted to two mastoid operations. Her head gets sore when she strains her eyes; even her teeth then ache. It hurts her to look at anything attentively. The tendons of some of the ocular muscles were scissored some

### CHART FOR DR. SWEET'S IMPROVED EYE LOCALIZER.

Made by Roentgen Manufacturing Co.

Surgeon F. Park Lewis Patient Barlow, John

Date May 2, 1910. No. 1402

Scale Full Size In m.m.

Size of body — by — by — m.m.

Location of body in ________ eye

| End | Back of Centre of Cornea | Horizontal Plane above | Horizontal Plane below | Vertical Plane temporal side | Vertical Plane nasal side |
|---|---|---|---|---|---|
| | | | | | |
| | | | | | |

Remarks—

March 9, 1909.

Rev B[illegible] 1910

eleven years ago. She has worn prisms for seventeen years. She has been treated for sinus-diseases. She has long had intense pains in her shoulders, and the like, ascribed to her "nerves" by her physicians. The list of her oculists includes a number of the most famous ophthalmologists. I found her wearing:

R.+S.0.50—C.1.75 ax.180°2°Prism B.O.)

L.+S.0.75—C.2.75 ax.180°1.5 Prism B.O.(

Fix, now, that correction in mind and compare it with what this woman's eyes plainly demanded, viz:

R.—S.0.25+Cyl.0.50 ax.75°=20|20+
L.+S.0.25—Cyl.2.75 ax. 5°=20|20+ Distance
R.+S.0.75 & Cyl.)
L.+S.1.25 & Cyl.) Near In bifocal spectacles.

Think of a right-eyed woman wearing such a right lens as that ordered for her! What could the abused visual mechanism do with such a glass except to turn the eye any way to be rid of it. *Hence the 19° of esophoria* I found present.

Under correct lenses, this woman's esophoria began lessening. Possibility of physiologic binocular vision had not been lost despite all that Nature had failed to do as regards the shape of the eyeballs, and despite all that man had been able to add to the difficulty and to the pathology. In a week the esophoria had been almost reduced to the desired normal, and the atrocious sufferings of a life were rapidly lessening. In view of the age of the woman the final complete cure, in so short a time, was astonishing. Usually the ominous age of 40 makes cure within a year very unusual. But before this year has fairly begun, and taught or advised by physicians, the patient usually will rush off to the Professor of Head-Surgery, or to the insane asylum, or the "Home," or to Mrs. Eddy, or to the German "Cure," and therefore remain forever uncured. The art of practising rational therapeutics in all such cases is the art of keeping patients patient until the old injuries have been healed by correct lenses.

When the age is 44 it will probably take nearer two years to effect a cure even in those with less thoroughly ruined eyes and nervous systems. Such a case is that of a man who came to me about two years ago. With the onset of presbyopia at 40 the usual rise or increase of morbid nerve symptoms (universally unknown and ignored, but of awful significance) began. In this patient's case *"petit mal"* appeared, soon degenerating into typical *"grand mal"* epileptic seizures with biting of the tongue, and the like, becoming more frequent as nothing neurological or ophthalmological was proved of avail. The oculists consulted gave him prisms, and changed the prisms frequently. I found him wearing, B.E.+Cyl.0.50 ax.90°. He has constant diplopia beyond near range due to an esophoria of 28° and hyperphoria of 3°. As usual his oculists had not ordered the highly desirable bifocals.

My prescription read as follows:

B.E.+Sph.0.37+Cyl.0.87 ax.90° Distance

B.E.+Sph.1.62 & Cyl. Near. In bifocal spectacles.

The patient returned to his far-distant home and I have not seen him since. In the few days of his stay the esophoria began to decrease, and the diplopia became less constant and noticeable. Fortunately, whatever else the patient may have done in his extremity and his despair, he did *not* follow the advice of some "subtemporal-decompression" operator, who, perhaps, jerks glasses off consulting patients and throws them vindictively into the corner. Instead of this, by an exhausting and burdensome correspondence, by encouragings, by exaction of constant reports, by fighting the quack epilepsy cures, by demanding consultations, by reports from other oculists (and keeping them from changing lenses)—I succeeded in holding my patient's confidence. He was too poor to make again the thousand-mile journeys to me. In a month he wrote of some lessening of the epileptic and other symptoms, especially the diplopia. In a year I secured the report of a local oculist saying that the esophoria was then only 12°; hopes began to mount. There was now no double vision. The reports said that the attacks were lessening in number and that the loss of consciousness was for a shorter time than ever before. Some six months ago the man's reports suddenly ceased. Gratitude ceases when the cure is complete. I hear from other patients who know him that there are no epileptic attacks now, and that he is perfectly healthy and happy.

## Figures and Tables

**Figure f1:**